# Toward a general and interpretable umami taste predictor using a multi-objective machine learning approach

**DOI:** 10.1038/s41598-022-25935-3

**Published:** 2022-12-16

**Authors:** Lorenzo Pallante, Aigli Korfiati, Lampros Androutsos, Filip Stojceski, Agorakis Bompotas, Ioannis Giannikos, Christos Raftopoulos, Marta Malavolta, Gianvito Grasso, Seferina Mavroudi, Athanasios Kalogeras, Vanessa Martos, Daria Amoroso, Dario Piga, Konstantinos Theofilatos, Marco A. Deriu

**Affiliations:** 1 Department of Mechanical and Aerospace Engineering, Politecnico di Torino, PolitoBIOMedLab, 10129 Torino, Italy; 2 InSyBio PC, 265 04 Patras, Greece; 3 Department of Innovative Technologies, Dalle Molle Institute for Artificial Intelligence, 6962 Lugano-Viganello, Switzerland; 4 Industrial Systems Institute, Athena Research Center, 265 04 Patras, Greece; 5 Faculty of Computer and Information Science, University of Ljubljana, 1000 Ljubljana, Slovenia; 6 Department of Nursing, University of Patras, 265 04 Patras, Greece; 7 Department of Plant Physiology, Institute of Biotechnology, University of Granada, 18011 Granada, Spain; 8 7hc srl, 00198 Rome, Italy

**Keywords:** Computational biology and bioinformatics, Molecular biology, Engineering, Mathematics and computing

## Abstract

The umami taste is one of the five basic taste modalities normally linked to the protein content in food. The implementation of fast and cost-effective tools for the prediction of the umami taste of a molecule remains extremely interesting to understand the molecular basis of this taste and to effectively rationalise the production and consumption of specific foods and ingredients. However, the only examples of umami predictors available in the literature rely on the amino acid sequence of the analysed peptides, limiting the applicability of the models. In the present study, we developed a novel ML-based algorithm, named VirtuousUmami, able to predict the umami taste of a query compound starting from its SMILES representation, thus opening up the possibility of potentially using such a model on any database through a standard and more general molecular description. Herein, we have tested our model on five databases related to foods or natural compounds. The proposed tool will pave the way toward the rationalisation of the molecular features underlying the umami taste and toward the design of specific peptide-inspired compounds with specific taste properties.

## Introduction

Umami taste is one of the five basic taste modalities and it is typically associated with the protein contents of foods. The term “umami” originates from a Japanese word that means “pleasant savoury taste”, “mouthfulness” or “delicious”^1^. Umami has been linked for several years to the taste of Asiatic traditional foods or cheese and it was recognized as the fifth basic taste modality—along with sweet, bitter, salty and sour—only in 2002 to describe a pleasant or glutamate-like taste^2^. Since the umami taste is commonly linked to the food protein content, it represents an interesting taste modality, especially for, but not limited to, food industries: considering the laboriousness of traditional experimental techniques, it is pivotal to develop fast, reliable and cost-effective methodologies able to predict the taste of food ingredients or general compounds with the ultimate goal of identifying and characterizing their chemical profile. Several experimental methods, including MALDI-TOF-MS and reversed-phase high-performance liquid chromatography (RP-HPLC) analysis, are widely used to identify and characterize peptides with umami sensory properties^3,4^. However, traditional experimental methods for characterizing and profiling from a chemical point of view the umami peptides are expensive, time-consuming, and arduous. In this context, the *in-silico* techniques have been pointed out as elicit methods to screen massive databases of compounds and retrieve specific information regarding their activity or properties through the employment of machine learning algorithms. Quantitative structure–activity relationships/quantitative structure–property relationships (QSAR/QSPR) methods aim at determining a relationship between the biological activity or the physicochemical property, respectively, and a set of descriptive features (descriptors) linked to the molecular structure of the investigated molecules^5^. In this regard, the guidelines defined by the Organization for Economic Co-operation and Development (OECD) indicate the strategies for the correct development and validation of robust QSAR models: (i) a defined endpoint; (ii) an unambiguous algorithm; (iii) a defined domain of applicability; (iv) appropriate measures of goodness-of-fit, robustness and predictivity; (v) a mechanistic interpretation, if possible^6^.

Regarding the in-silico prediction of taste based on the molecular structure of compounds, a lot of advancements have been accomplished^7^. For example, several publications deal with the prediction of the sweet taste^8–14^, the bitter taste^15–22^, and the bitter/sweet dichotomy^23,24^. However, as far as the authors know, there are few attempts made by the scientific community to predict the umami taste, which are represented by the iUmami-SCM^25^ and the UMPred-FRL^26^ predictors. The iUmami-SCM tool predicts the umami/non-umami taste of peptides based on their primary amino acid sequence employing a scoring card method (SCM) in conjunction with the propensity scores of amino acids and dipeptides. For its design, this tool is limited to the prediction of only peptides, which however represent the candidate par excellence of umami taste. Another effort again focused on umami peptide identification is the UMPred-FRL tool, which demonstrates a higher feature discriminative capability to capture the key information about umami peptides and superior performance compared to the iUmami-SCM. However, a method for screening databases of general molecules or predicting the taste of peptides with small chemical deviation from their original structures is needed to pinpoint the major physio-chemical properties related to the occurrence of the umami taste and allow the identification of umami-related compounds from bigger pools of potential compounds. The present work is therefore based on these premises and is devoted to developing an efficient tool to predict the umami/non-umami taste of query molecules based on their chemical structure described using the standard SMILES representation and commonly employed molecular descriptors. An ensemble dimensionality reduction and classification techniques were used to train and test the umami taste prediction model, minimizing the number of physicochemical features used as inputs and allowing the identification of the most important features related to the umami taste. The minimization of the inputs makes the prediction models simpler, reducing thus the risk of overfitting, and enables the incorporation of the prediction models in a web interface enlarging the ensemble of possible end-users. The developed tool, named VirtuousUmami, paves the way toward the possibility of analyzing different types of compounds and rationalising the chemical-physical characteristics at the basis of umami taste perception to design new ingredients and molecules with specific taste properties.

## Results

### Dimensionality reduction

**Figure 1:**
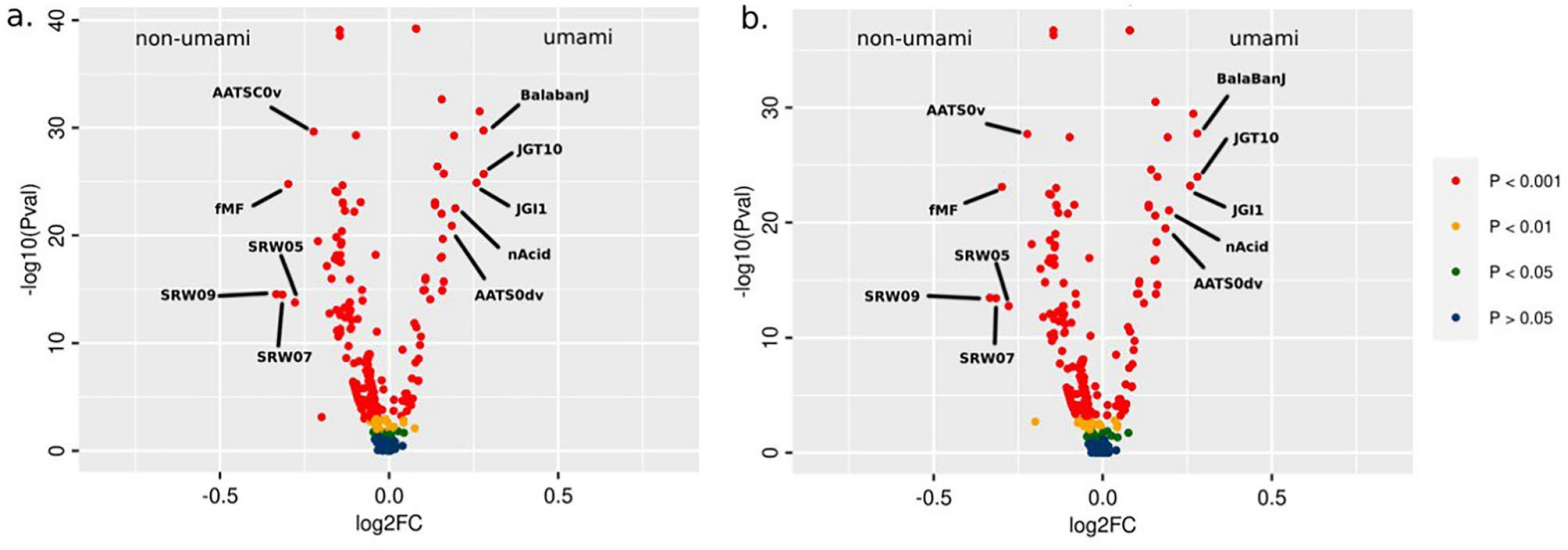
Volcano plots of the statistical analysis of the descriptors on the umami versus non-umami samples for the training set (**a**) with the standard limma eBayes method using p-values and (**b**) with correction of p-values using the Benjamini–Hochberg FDR adjustment method to calculate q-values. Only the 5 most upregulated and 5 most downregulated features are labelled for the sake of clarity.

As described in the “Methods” section, the statistical analysis to reduce the number of employed molecular descriptors was performed on the training set with the limma eBayes method^27^. Moreover, the correction of p-values for multiple testing to get q-values was applied using the Benjamini–Hochberg FDR adjustment method^28^. Setting the q-value threshold to 0.05, we identified 324 statistically significant differentiated features. This analysis is shown in Fig. 1 in a volcano plot representation with the log2 of the Fold Change (log2FC) on the x-axis and the negative value of the logarithm of the p- or q-values on the y-axis. The log2FC was calculated for each feature by applying the log base 2 to the ratio between the average value of the feature for the umami class and the average of the non-umami class. P-values (Fig. 1a) and q-values (Fig. 1b) less than or equal to 0.05 denoted statistically significant differences between umami and non-umami samples, whereas positive log2FC values denote upregulated features, i..e features with higher values in umami than non-umami compounds, and negative log2FC values indicate downregulated features. In this view, the most informative features in the volcano plots are located at the top and far from the zero value of the x-axis. The detailed list of the prioritized molecular descriptors is available in the GitHub repository (https://github.com/lorenzopallante/VirtuousUmami) within the “data” folder (“umami_prioritized_list_of_descriptors.csv”).

### Model performance

**Figure 2:**
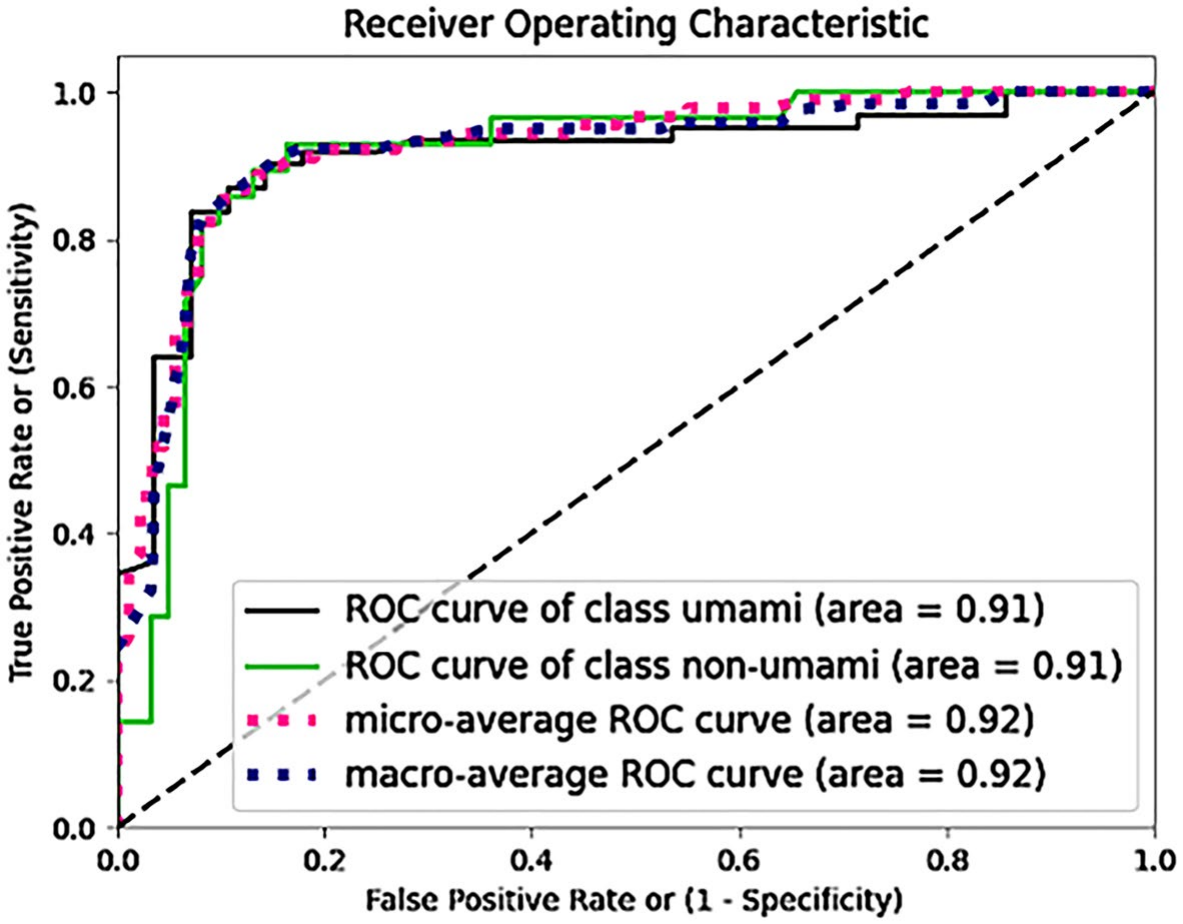
Receiver operating characteristic curve of the umami versus non-umami classification.

We developed 5 different SVM models with a specific number of selected features and support vectors (see also Table 1). After accessing the performance of the single SVM models (Table S2), we developed 10 ensemble models (EMs) by taking all the possible combinations between the SVM models (1 and 2; 1 and 3; 2 and 4; etc..) and evaluated the relative performance (Table S3). The EM_3–5_, i.e. the ensemble model created combining SVM models 3 and 5, achieved the best performance and was selected as the final model. A summary of the model performance for the EM_3-5_ is reported in Table 1 and the relative ROC curves are represented in Fig. 2.

**Table 1:** Summary of model performance using the ensemble model EM_3-5_ obtained from the combination of SVM models 3 and 5.

	ACC	Spec	Sens	F1	F2	AUC
Training	99.79% ± 0.01	99.59% ± 0.02	100% ± 0.009	99.79% ± 0.01	99.92% ± 0.01	1 ± 0.007
tenfold CV	95.86% ± 1.89	96.70% ± 2.91	95.07% ± 1.06	95.73% ± 1.81	95.28% ± 0.88	0.96 ± 0.02
Test	87.64%	91.80%	78.57%	79.31%	80.99%	0.85

For the training set and the tenfold cross-validation mean values and standard deviations are presented. The test set comprises the 90 left-out samples not used for training.

### Feature importance

The selected features on which the predictions rely are 12 and include ATSC1m, Xch_6d, Mi, SaaCH, SMR_VSA1, JGI1, FilterItLogS, JGT10, AATSC0m, AATSC0v, Mp, fragCpx. The selected features are summarized in Table 2 also reporting the level of importance evaluated with the calculation of the SHAP values^29^. The distributions of the 12 features for the umami and non-umami samples are represented in Figs. S1 and S2.

**Table 2:** Features selected according to the best model.

ID	Name	Module class	Description	SHAP importance
1	ATSC1m	Autocorrelation	Centered moreau-broto autocorrelation of lag 1 weighted by mass	0.1090
2	AATSC0m	Autocorrelation	Averaged and centered moreau-broto autocorrelation of lag 0 weighted by mass	0.0821
3	AATSC0v	Autocorrelation	Averaged and centered moreau-broto autocorrelation of lag 0 weighted by vdw volume	0.0416
4	JGI1	TopologicalCharge	1-ordered mean topological charge	0.0331
5	JGT10	TopologicalCharge	10-ordered global topological charge	0.0323
6	SMR_VSA1	MoeType	MOE MR VSA Descriptor 1 (− inf < x < 1.29)	0.0296
7	Mi	Constitutional	Mean of constitutional weighted by ionization potential	0.0264
8	FilterItLogS	LogS	Filter-it™ LogS	0.0176
9	Mp	Constitutional	Mean of constitutional weighted by polarizability	0.0174
10	SaaCH	Estate	Sum of aaCH	0.0170
11	Xch-6d	Chi	6-ordered Chi chain weighted by sigma electrons	0.0122
12	fragCpx	FragmentComplexity	Fragment complexity	0.0083

SHAP values represent the contribution of each feature to the prediction. The greater the value, the higher the contribution.

Among the 12 selected features, the most frequent descriptor class represents internal autocorrelation properties (ATSC1m, AATSC0m, AATSC0v), calculated by the so-called Autocorrelation of a Topological Structure (ATS), which describes how a property is distributed along with the topological structure. In particular, the autocorrelation descriptors were computed using the Moreau-Broto autocorrelation weighted by mass (ATSC1m and AATSC0m) or Van der Waals volume (AATSC0v). Interestingly, the three autocorrelation properties were also retrieved among the first eight prioritized features from the initial univariate filtering. The Xch-6d descriptor belongs to the Chi descriptors family, which are topological indexes based on the molecular connectivity approach^30^. Molecular connectivity methods quantify molecular structures based on the topological and electronic characters of the atoms in the molecule. The molecule is represented by the hydrogen-suppressed graph (molecular skeleton) and the key feature in the quantitation of the graph is the characterization of the atom in the molecular skeleton. The molecular graph may be decomposed into fragments called subgraphs, such as a skeletal bond, a pair of adjacent bonds, etc., that determine the possibility of defining different orders of the indexes: thus, the order of the Chi index is the number of edges in the corresponding subgraph. Mi and Mp are instead the mean of constitutional properties, i.e. the ionization potential and the polarizability, respectively. SaaCH descriptor is an Electropological State (Estate) index^31^, which is a combination of electronic, topological and valance state information. In particular, this descriptor is calculated for specific atoms types: in this case, SaaCH stands for the sum of E-state indices for the CH in an aromatic ring. The SMR_VSA1 descriptor is a MOE type descriptor that uses a combination of the Wildman-Crippen Molar Refractivity (MR)^32^, which is a measure of the total polarizability of a mole of a substance, and the Van der Waals surface area contribution. Two other descriptors, namely JGI1 and JGT10, deal instead with the compounds’ topological charge considered at the first and 10^th^ orders, respectively. FilterItLogS descriptor is derived from a program designed for filtering out molecules with unwanted properties. The program is packaged with several pre-programmed molecular properties that can be used for filtering, including (i) physicochemical parameters, such as logP, topological polar surface area criteria, number of hydrogen bond acceptors and donors, and Lipinski’s rule-of-five; (ii) graph-based properties, including ring-based parameters and rotatable bond criteria; (iii) selection criteria through smarts patterns; (iv) Similarity criteria; (v) three-dimensional distances between user-definable fragments (https://github.com/silicos-it/filter-it). Finally, the fragCpx descriptor is a fragment complexity descriptor which is calculated as:

1 $$\mathrm{fragCpx}=\left|{B}^{2}-{A}^{2}+A\right|+\frac{H}{100}$$

where A is the number of atoms, B is the number of bonds, and H is the number of heteroatoms^33^.

Hierarchical clustering of the selected features allows grouping of the 12 features in three subgroups, i.e. (i) AATSC0v, ATSC1m, Mp, (ii) fragCpx, SMR_VSA1, AATSC0m, SaaCH, Xch-6d, (iii) JGI1, JGT10, Mi, FilterItLogS (see also Fig. S3).

**Figure 3:**
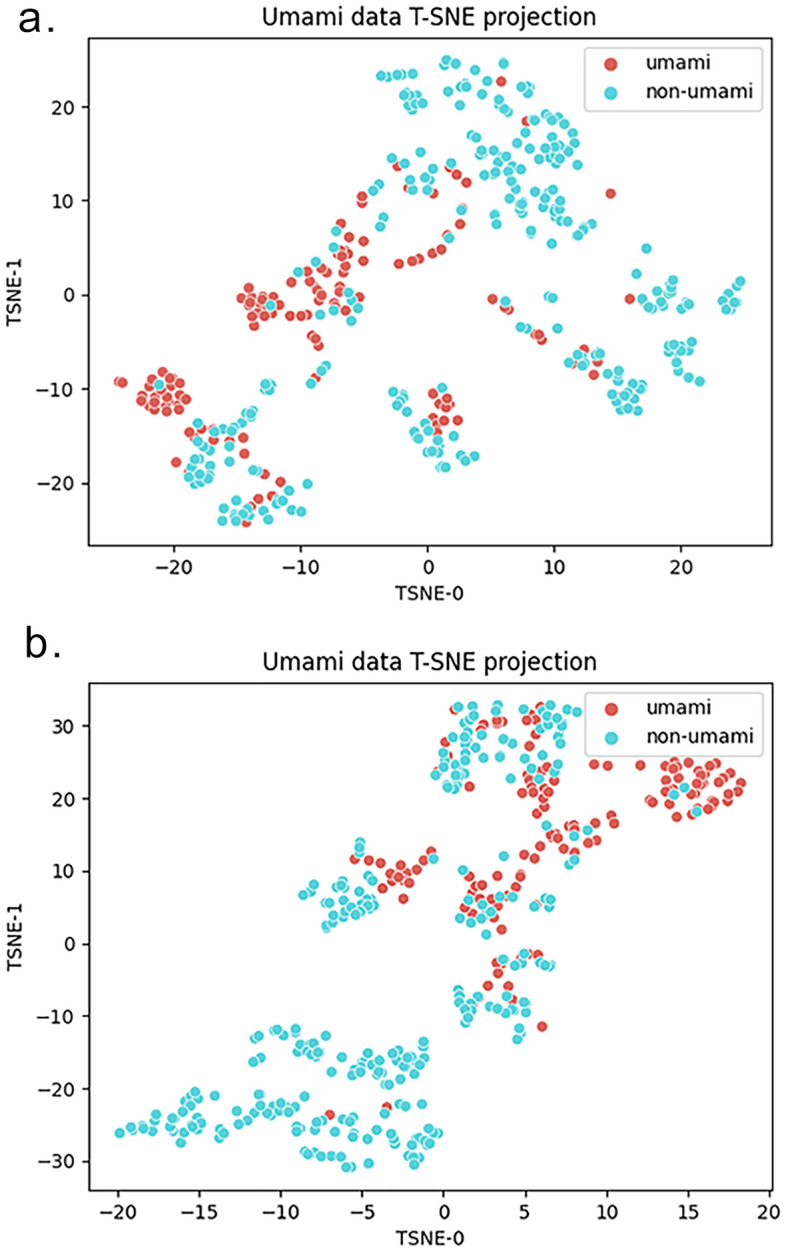
A tSNE applied to the umami and non-umami samples for the whole dataset taking into account (**a**) all molecular descriptors (1613 features) and (**b**) the best 12 selected descriptors derived from the feature selection process. The selected feature subset (**b**) results in a remarkably better ability in discriminating between umami and non-umami compounds.

To represent the dataset’s chemical space and underline the role of the feature importance analysis in simplifying the discrimination between the umami and non-umami, we used the tSNE dimensionality reduction technique^34^ on the starting dataset taking into account all descriptors and only the best 12 above-mentioned features (Fig. 3).

### Applicability domain (AD)

**Figure 4:**
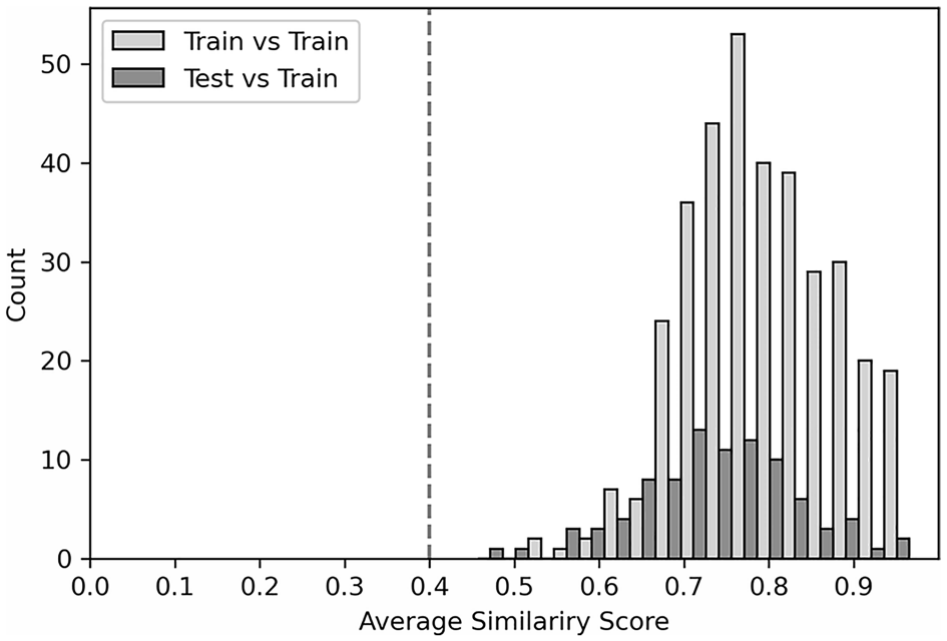
Histograms of average similarity scores of training and test sets. The average similarity score is derived by averaging the Tanimoto similarity score between the five most similar compounds in the training set. The light grey histogram represents the distribution of the average similarity scores for all the compounds composing the training set, whereas the dark grey histogram the distribution for the test set. The lower limit of the above-mentioned distributions allows for determining the similarity threshold of the applicability domain.

To effectively define the applicability domain (AD) of the model, we evaluated the average similarity scores of both training and test sets compared to the training sets fingerprints, as described in the “Methods” section. The analysis reported in Fig. 4 allowed us to establish a correct average similarity threshold (i.e. 0.4) to effectively determine if a query compound falls inside or outside the AD based on the average similarities of the employed dataset. In particular, if the average similarity score of a query compound is below the imposed threshold, then the query compound is considered outside the AD; otherwise, the compound is considered within the AD.

### External datasets

The external datasets, i.e. FlavorDB, FooDB, NPAtlas, PhenolExplorer and PhytoHub, were processed as reported in the “Methods” section. Results are summarised in the following.

1. *FlavorDB* After removing 380 compounds with issues from the ChEMBL structure pipeline, we got 2599 compounds. Checking the AD of the umami model, we pointed out that only 0.92% (24/2599) of the FlavorDB molecules are inside the umami AD. Our model predicted 9 of the 24 compounds (36%) as umami.
2. *FooDB* Among the 70 k chemicals included in the dataset, we preserved 69,309 molecules after removing missing SMILES, duplicate compounds, and molecules with structure errors according to RDKit import functionality and high issues based on the ChEMBL Structure Pipeline. Only 1.09% (757/69,309) of these molecules fall inside the AD of the model. 48% of these molecules (366/757) were then predicted as umami.
3. *Natural product atlas* After running the ChEMBL structure pipeline, we preserved 32,491 compounds. 1.52% (495/32,491) of the molecules are inside the AD of the model and 17.3% of these molecules (86/495) were then predicted as umami.
4. *PhenolExplorer* We first removed 3 compounds with issues according to the ChEMBL structure pipeline, obtaining 489 compounds. According to the AD, only 0.61% (3/489) of the PhenolExplorer molecules are inside the AD of the model. None of these molecules was predicted as umami.
5. *PhytoHub* From the original dataset of 2110 compounds, we removed compounds with missing SMILES (294) or high issue scores from the ChEMBL structure pipeline (70), resulting in a database of 1746 molecules. Only a small percentage, i.e. 1.03% (18/1746), of the PhytoHub molecules are inside the applicability domain of the umami model. Just one molecule among the 18 compounds (5.5%) was predicted as umami.

Predicted umami compounds for each of the external DBs are available in the GitHub repository (https://github.com/lorenzopallante/VirtuousUmami) within the “data” folder.

### Virtuous umami platform

The developed umami predictor was embedded into a web-based interface, namely the Virtuous Umami platform (http://195.251.58.251:19009/#/virtuous-umami). This is a graphical, user-friendly interface for running analyses for chemical compounds expressed in various notations, including SMILES, FASTA format, InChI, SMARTS or PubChem compound name. If the PubChem name is provided by the user, the algorithm queries the database for the requested compound retrieving the relative canonical SMILES to run the umami prediction model. The platform is built using open-source programming solutions and is divided into two main components, i.e. the front-end and the back-end. The front-end is the part of the application that is visible to the users and runs on their devices. It provides them with the option to type compounds directly to an input field or to upload a text file containing each compound in a different line. After the analysis takes place, the results are presented in a tabular form that reports the query compound SMILES, its 2D molecular representation, the verification of the domain of applicability (True/False), the result of the umami prediction (Yes/No) and two buttons allowing the user to download the databases collecting all the calculated Mordred molecular descriptors or the best 12 on which the prediction relies. For developing the front-end, the Ionic framework was selected because it offers a wide variety of UI components that can be used to create user-friendly applications suitable both for browsers and mobile devices. The second main component, the backend, consists of a web service that runs on the cloud and is implemented using the lightweight yet powerful Flask micro-framework. It is responsible for receiving the input sent by the front-end, running the Virtuous Umami Analyser and returning the results to the front-end. To enable the aforementioned exchange of information, it provides a RESTful API that accepts and transmits data in the form of JavaScript Object Notation (JSON).

## Discussion

Machine Learning methods have proven to play a key role in the development of prediction tools and digital support systems in a variety of application areas, including nutrition and agri-food research^35–42^. In this context, here, we developed a novel machine-learning-driven umami taste predictor, named VirtuousUmami, to identify umami/non-umami compounds based on the SMILES representation. The classification model was generated with the hybrid combination of heuristic optimization and nonlinear machine learning classification methods, allowing both an unbiased and an optimized selection of the classification method and its parameters.

Starting from the UMP442 database^25^, which collects 442 peptides, we used the Mordred molecular descriptors to obtain the features: the Mordred library is open source and demonstrated high computational efficiency and stability^43^. Moreover, we decided to only compute 2D molecular descriptors to avoid the impact of compound optimization and parameters related to the three-dimensional properties of molecules. The exhaustive list of the employed Mordred descriptors is available at https://mordred-descriptor.github.io/documentation/master/descriptors.html. The 2D Mordred descriptors provide information on compounds, such as basic information about molecules (molecular weight, number of individual types of atoms, types of bonds, degree of hybridization, spectral diameter, detour index, number of hydrogen donors and acceptors, molecular distance edge between different types of atoms, polarizability of atoms and bonds, and topological polar surface) and other features derived from symbolic representations (Zagreb index, adjacency matrix descriptors, Moreau–Mroto descriptors, Moran coefficients, Geary coefficients, and descriptors describing the Burden matrix and Barysz matrix)^44^. It is worth mentioning that other previous works successfully obtained good results in the field of taste prediction using only 2D molecular descriptors^9,24^: this represents a great step forward since 2D molecular descriptors are less expensive from a computational point of view and not affected by variations in the three-dimensional molecular structures.

Since the number of molecular descriptors (1613) was extremely higher than the number of compounds in the dataset (442), the limma eBayes statistical analysis was employed to reduce the total number of descriptors to 324, boosting the performance of the subsequent refined model. The best performance obtained from an ensemble of models in terms of accuracy (ACC), specificity (Spec), and sensitivity (Sens) scores are in good agreement with the state of the art^25,26^. In this context, to provide a comparison with previously developed umami prediction tools, iUmami-SCM^25^ and UMPred-FRL^26^ were assessed with the VirtuousUmami test set (Table S4). Comparing the evaluated metrics, the three algorithms showed overall similar performance, in terms of accuracy (ACC), specificity (Spec), sensitivity (Sens), F1 and F2 scores with all values roughly in the range of 80%-90%. Moreover, one of the major novelties of VirtuousUmami relies on its generalizability and applicability. In greater detail, its ability to process several types of molecular structure notations, including SMILES, FASTA, InChI, SMARTS or PubChem name allows screening for any type of compound, thus opening up the possibility to screen a wide range of molecular databases for detecting umami compounds. In this context, we employed the VirtuousUmami predictor on five different external databases related to food or natural compounds, i.e. FlavorDB, FoodDB, Natural Product Atlas, PhenolExplorer and PhytoHub, highlighting compounds with umami character. Another important advantage of the proposed model relies on its explainability.

The usage of general molecular descriptors from the Mordred library and the employment of dimensionality reduction algorithms, such as statistical significance analysis and the SHAP feature importance, allowed the definition of a reduced number of interpretable features on which the model relies: in this case, the best model was able to achieve the above classification scores with only 12 features. Figure 3 graphically remarks on the importance of the feature selection procedure: the selected feature subset (Fig. 3b) can discriminate remarkably better between umami and non-umami taste if compared to the tSNE analysis taking into account all the descriptors (Fig. 3a). Despite the remarkable reduction in the number of features, it still remains complex to intuitively highlight the chemical and physical properties of umami/non-umami compounds related to the 12 most important features. In this sense, it will be very important in future studies to be able to use simpler descriptors in order to improve the explainability of the model. The definition of a small subset of important molecular features profoundly differentiates the approach proposed by previously developed methods, such as iUmami-SCM^25^ and the UMPred-FRL^26^, which based their predictive models only on the peptide sequences. While the possibility of optimising a predictive model on the peptide sequence alone is a great advantage in terms of model simplification, it also makes it very complicated to pinpoint the chemical-physical characteristics underlying molecules' properties and thus explain the model prediction coming from the machine learning black box.

Moreover, following the guidelines defined by the Organization for Economic Co-operation and Development (OECD)^6^, we also developed an applicability domain (AD) to provide information regarding the reliability of the prediction. From this analysis, we pointed out that the distribution of the average similarities of training and test sets are similar in shape, denoting that the dataset is homogeneous and correctly repartitioned between training and test sets (Fig. 4). The distribution of the average similarity scores towards elevated values suggests a high similarity among the compounds composing the dataset and, therefore, a quite narrow chemical space of the umami database. In this context, the development of an applicability score ensures reliable predictions for compounds within the above-mentioned domain. The above-mentioned limited spectrum is a direct consequence of the limited number of umami/non-umami compounds available from previous literature and composing our training dataset. In particular, the limited number of positive samples in the dataset (only 28 umami compounds in the test set and 112 in the training set) limits the accessible chemical space of the umami samples in the training phase and the subsequent prediction ability of the model on the positive class, causing differences in the sensitivity scores in the test (78.6%) and training (roughly 95.1%) sets. In this case, the model sensitivity was particularly affected by the considerably few positive samples in the test set. The reduced number of compounds in the employed dataset, i.e. UMP442, is an important limitation of the present as well as previously developed umami predictors: likely, a larger size of the umami dataset will result in higher performance. Nevertheless, it is worth mentioning that the VirtuousUmami sensitivity (78.6%) is in the agreement or higher than the ones of UMPred-FRL^2^ (78.6%) and iUmami-SCM^1^ (71.4%) respectively, when tested against the VirutousUmami test set (see also Table S4). In conclusion, future extensions in available experimental data concerning umami/non-umami compounds will be pivotal to enlarging the investigated chemical space and the applicability of ML-driven methodologies, such as VirtuousUmami.

Finally, the development of a user-friendly web interface (http://195.251.58.251:19009/#/virtuous-umami) stems from the idea of making the umami prediction model usable even for users not experienced or familiar with the use of technical python codes (also available in the GitHub repository at https://github.com/lorenzopallante/VirtuousUmami).

In summary, VirtuousUmami will be a powerful tool to fastly screen any compound database for the discovery of a wide range of candidate compounds with potential umami sensory properties. In a broader view, it is worth mentioning that the method developed within this work is fully generalizable to the prediction of other taste sensations since it is based on the SMILES format, a standard description and widely used by the scientific community: the present tool, therefore, lays the foundations for the creation of a general tool for the prediction of the five basic tastes.

## Methods

### Data curation

For an effective comparison with previous literature dealing with umami taste predictors, the UMP442 database, also used for iUmami-SCM^25^ and UMPred-FRL^26^ predictors, was employed. The UMP442 dataset is freely accessible from GitHub https://github.com/Shoombuatong/Dataset-Code/tree/master/iUmami) and collects 442 peptides (140 umami and 302 non-umami): umami molecules are gathered from previous literature^1,45–49^ and the BIOPEP-UWM database^50^, whereas non-umami peptides are the bitter peptides from the positive set of the BTP640 database^51^ (see also Table S5). The peptides were gathered using their amino acid sequences and then converted into their SMILES representation using the RDKit package (http://www.rdkit.org). Then, they were processed with the ChEMBL Structure Pipeline^52^ (https://github.com/chembl/ChEMBL_Structure_Pipeline) to highlight possible issues in the retrieved molecular structure and to standardise the SMILES representation for the entire dataset. The latter protocol runs a molecule checker on the compound structure, standardizes chemical structures and generates the parent molecule representation based on a set of predefined rules.

Among 442 umami (140) and non-umami (302) peptides available in the UMP442 dataset, 352 ligands were used for training. The remaining 90 peptides were used for external testing to examine the generalization properties of the trained models. Of the 352 training samples, 240 were non-umami samples, and 112 were umami samples. Because there is an imbalance in the total number of samples of the two classes, we oversampled the umami class, creating synthetic data to boost the umami class. These synthetic data were created by selecting random samples from the umami class and duplicating them, a method of random oversampling for the minority class. The resulting training dataset had 240 non-umami samples and 240 umami samples. Of the 90 testing samples, 62 were non-umami samples, and 28 were umami samples. The summary of the final dataset is also reported in Table S6.

### Molecular descriptors and dimensionality reduction

The calculation of the features for each one of the molecules was achieved using 1613 2D Mordred descriptors. The dataset was preprocessed to be used as input to the machine learning model. In particular, features with a high percentage of missing values (> 30%) were filtered, while the remaining missing values were imputed using the kNN-impute method with k = 20^53^. Then, data were arithmetically normalized to the interval of [0–1]. Given the huge number of total features, i.e. 1613, compared to the size of the training dataset, an initial univariate filtering approach was deployed. The statistical analysis was performed on the umami vs non-umami peptides of the training set with the limma eBayes method^27^, and correction of p-values for multiple testing was performed using the Benjamini–Hochberg FDR adjustment method^28^ to calculate q-values. For both p- and q- values a threshold of 0.05 was applied. We used also four different feature selection methods, i.e. the Wilcoxon Rank Sum Test^54^, kBest, JMIM^55^ and MRMR^56^, to further reduce the dimensionality of the training dataset. These methods were iteratively tested using an in-house evolutionary optimization algorithm (50 individuals and 100 generations) which identified the best combination of feature selection techniques among the above-mentioned alternatives. The results of these methods are used at every generation of the evolutionary algorithm for every individual to reduce the features in the training process. In this way, we are confident that at each run we select the most important features for our problem.

Data preprocessing, statistical analysis and the generation of additional plots, such as ROC curves and bean plots, were performed using the InSyBio Biomarkers Tool (see also the reference Manual for further details at https://www.insybio.com/biomarkers.html).

### Model construction and performance evaluation

The classification models were generated with the hybrid combination of heuristic optimization and nonlinear machine learning classification methods incorporated in the InSyBio Biomarkers tool (https://www.insybio.com/biomarkers.html). Specifically, we used an ensemble dimensionality reduction technique employing a heuristic multi-objective Pareto-based evolutionary optimization algorithm^57^ to (a) identify the optimal feature subset to be used as input to the classifiers, (b) select the most appropriate classifier among Support Vector Machines (SVM) and Random Forests and (c) select the optimal parameters for the classifier, namely C and gamma of SVM and number of trees for Random Forests. This approach allows both an unbiased and an optimized selection of the classification method and its parameters. The multi-objective Pareto-based approach was deployed to handle the multiple objectives of maximization of predictive performance, minimization of selected features and simplicity of the classification model, revealing all the non-dominated solutions of the above-stated optimization goals. The weights used for the goals were Selected Features Number Minimization 5, Accuracy (ACC) 10, F1 score 5, F2 score 1, Precision (PRC) 1, Recall (REC) 10, ROC-AUC (AUC) 1, Number of SVs or Trees Minimization 1, which enable better handling of the imbalanced nature of our classification problem. The outcomes are multiple models performing equally well (namely, the Pareto set of optimal solutions) on the user-defined goals. After having defined the best models in terms of performance metrics, we developed ensemble models (EMs) to further improve the prediction performance. In greater detail, an ensemble model is built by combining two different single models: the final prediction probabilities of the ensemble model for the positive and negative classes is the average of the prediction probabilities coming from the two combined models. The final predicted class is therefore the one with the highest probability score.

A population of 50 individuals was used for the evolutionary algorithm and a maximum number of 100 generations was used as the termination criterion. To deal with the stochastic nature of the proposed algorithm, five different runs were conducted and the results presented are the average performance of these runs. Convergence of the algorithm (average performance less than 5% different to best performing individual) was noted after 30 generations for each independent run demonstrating that the maximum number of generations used was adequate for this problem. Additional parameters of the evolutionary algorithm were set to their default values as suggested by the InSyBio Biomarkers tool user manual (arithmetic crossover probability: 0, mutation probability: 0.01, two-point crossover probability: 0.9). Stratified tenfold cross-validation was used to train and test the prediction models. To deal with the class-imbalanced nature of our data, in each cross-validation iteration, we applied random oversampling of the minority class in the 9 folds which were used to train the models. Further details on the implementation of the trained models and a summary of the performance metrics used are available in the Supplementary Information.

### Applicability domain

In the present work, following the guidelines defined by the Organization for Economic Co-operation and Development (OECD)^6^, we developed an applicability domain (AD) to provide information regarding the reliability of the prediction. We used an average-similarity approach already employed in previous recent literature in the taste prediction field^11,17^. More in detail, the AD was built as follows: (i) the Morgan Fingerprints (1024 bits, radius 2) were calculated using RDKit for all the compounds in the training set; (ii) a similarity score was then evaluated between each molecule in the training and test sets and the previously-defined fingerprints using the Tanimoto similarity index from RDKit; (iii) then the average similarity score was computed by averaging the similarity scores of the 5 most similar couple of compounds. The distribution of the average similarity scores for the training and test sets was used to identify a similarity threshold to discriminate between query compounds inside or outside the domain of applicability of the developed model. The AD check is performed every time before running the model to assess the reliability of the prediction and the output of the AD control is given to the user.

### External datasets

Several external datasets have been considered for testing the usability of the developed umami predictor. In particular, we chose some databases related to foods or natural products:

1. *FooDB* (https://foodb.ca/) is the world’s largest and most comprehensive resource on food constituents, chemistry and biology (more than 70 k compounds).
2. *FlavorDB* (https://cosylab.iiitd.edu.in/flavordb/) comprises 25,595 flavour molecules. For the present work, we considered only 2939 molecules related to natural ingredients.
3. *PhenolExplorer* (http://phenol-explorer.eu) collects a comprehensive database of polyphenols contained in foods. We considered only compounds having composition data (SMILES), i.e. 489 compounds.
4. *Natural Product Atlas* (https://www.npatlas.org/) includes microbially-derived natural products published in peer-reviewed primary scientific literature. We downloaded 32,552 natural compounds.
5. *PhytoHub* (https://phytohub.eu/) is a freely available electronic database containing detailed information about dietary phytochemicals and their human and animal metabolites. We downloaded 2110 compounds.

Each database was first checked for missing SMILES or data, standardised with the ChEMBL Structure Pipeline and, finally, the Mordred descriptors were calculated as done for the starting umami/non-umami dataset. Before running the model prediction, each dataset was screened to access the portion inside the model applicability domain and the prediction was then performed only in the above-mentioned portion.

## Supplementary Information

Supplementary Information.

## Data Availability

The established prediction model, together with supplementary data, is publicly released at https://github.com/lorenzopallante/VirtuousUmami and implemented into a user-friendly web interface (http://195.251.58.251:19009/#/virtuous-umami).
