# Supplementary material for: Toward a general and interpretable umami taste predictor using a multi-objective machine learning approach
Source: Sci Rep. 2022 Dec 16;12:21735. doi: 10.1038/s41598-022-25935-3 (PMC9758219; doi:10.1038/s41598-022-25935-3)
Supplement: Supplementary file 1 — Supplementary Information. [file 41598_2022_25935_MOESM1_ESM.pdf]

Supplementary Information

Results - Model Construction and Performance

The evaluation functions of maximization of predictive performance, minimization of selected features and simplicity of the classification model, which were used for guiding the optimization process are the following:

- Selected Features Number Minimization (SFNM):

$$SFNM = \frac{1}{1 + Number\ of\ selected\ features}$$

- Accuracy (ACC):

$$ACC = \frac{Tp + Tn}{Tp + Fp + Tn + Fn}$$

  where Tp represents the true positives, Tn the true negatives, Fp the false positives and Fn the false negatives.

- Precision (PRC):

$$PRC = \frac{Tp}{Tp + Fp}$$

- Recall (REC):

$$REC = \frac{Tp}{Tp + Fn}$$

- F1 Score (F1):

$$F1 = \frac{2 * \mathrm{PRC} * \mathrm{REC}}{\mathrm{PRC} + \mathrm{REC}}$$

- F2 Score (F2):

$$F2 = \frac{5 * \mathrm{PRC} * \mathrm{REC}}{4 * \mathrm{PRC} + \mathrm{REC}}$$

- ROC-AUC: Area Under the Receiver Operating Characteristic curve of Sensitivity/Specificity

- Number of SVs or Trees Minimization: Number of Samples in Training Set/Number of Support Vectors of the trained Support Vector Regression Problem

We developed 5 different models summarised in Table S1.

Table S1. Summary of the 5 developed models, including the number of support vectors in the SVM implementation and the number and type of features selected by each model.

Model	***#Support Vectors***	***Selected Features***
**1**	340	7: AATSC0m, Mp, Mi, FilterItLogS, SMR_VSA1, JGI1, JGT10
**2**	482	7: AATSC0m, Mi, SaaCH, fragCpx, FilterItLogS, VSA_EState7, JGI1
**3**	148	8: ATSC1m, Xch_6d, Mi, SaaCH, SMR_VSA1, JGI1, FilterItLogS, JGT10
**4**	340	10: ATSC1Z, AATSC0m, Mp, Mi, SaaCH, fragCpx, FilterItLogS, SMR_VSA1, JGI1, JGT10
5	148	8: AATSC0m, AATSC0v, Mp, Mi, SaaCH, fragCpx, FilterItLogS, JGI1

The model performance was evaluated on the test set for all models (Table S2).

Table S2. Performance of the 5 SVM developed models.

Model	***ACC***	***Spec***	***Sens***	***F1***	***F2***	***AUC***
**1**	73%	93.44%	28.57%	40%	32.26%	0.61
**2**	77.53%	95.08%	39.29%	52.38%	43.65%	0.67
**3**	85.39%	90.16%	75%	76.36%	75.54%	0.83
**4**	73%	93.44%	28.57%	40%	32.26%	0.61
5	86.52%	90.16%	78.57%	78.57%	78.57%	0.84

To improve the predictor's performance, ten ensemble models (EMs) were built by combining two different SVM (1 and 2; 1 and 3; 2 and 4; etc..) out of the five ones developed in this work. A comparative performance analysis highlighted EM_{3-5} (combination of SVM models 3 and 5) as the best ensemble model (Table S3)

Table S3. Performance of the ensemble models (EMs) optimised by combining the 5 SVM models. The ensemble model EM_{3-5} (combination of SVM models 3 and 5) achieved the best performance.

EM	***ACC***	***Spec***	***Sens***	***F1***	***F2***	***AUC***
**EM_{1-2}**	77.5%	93.44%	42.86%	54.55%	46.88%	0.68
**EM_{1-3}**	84.27%	88.52%	75%	75%	75%	0.82
**EM_{1-4}**	73.03%	93.44%	28.57%	40%	32.26%	0.61
**EM_{1-5}**	85.39%	88.52%	78.57%	77.19%	78.01%	0.84
**EM_{2-3}**	86.52%	90.16%	78.57%	78.57%	78.57%	0/84
**EM_{2-4}**	77.57%	93.44%	42.86%	54.55%	46.88%	0.68
**EM_{2-5}**	86.52%	90.16%	78.57%	78.57%	78.57%	0.84
**EM_{3-4}**	84.27%	88.52%	75%	75%	75%	0.82
**EM_{3-5}**	**87.64%**	**91.80%**	**78.57%**	**79.31%**	**80.99%**	**0.85**
**EM_{4-5}**	85.39%	88.52%	78.57%	77.19%	78.01%	0.84

Results - Feature Importance

The distributions of the 12 most significant features on which the prediction relies are represented in Figure S1 and Figure S2.

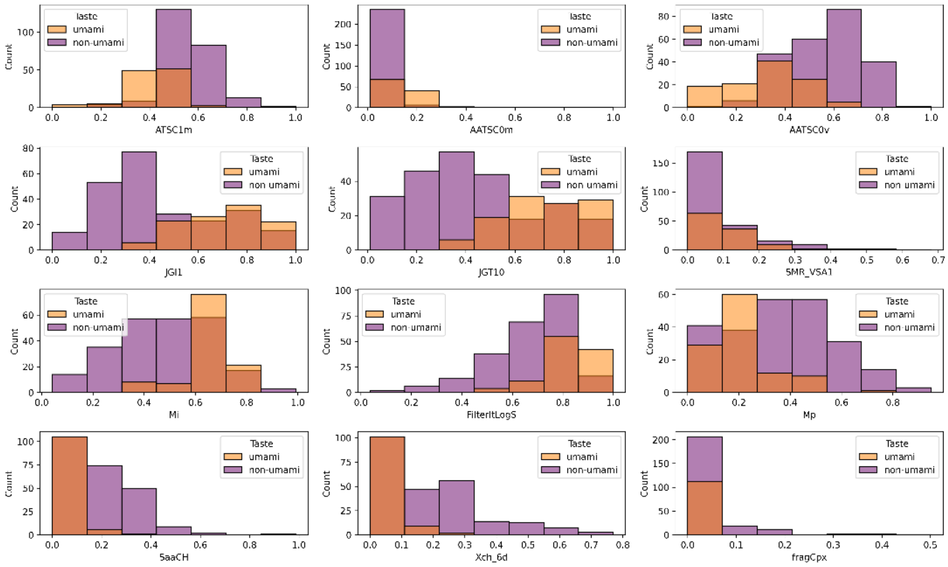

Figure S1. Distribution of the umami and non-umami data for the 12 most significant features

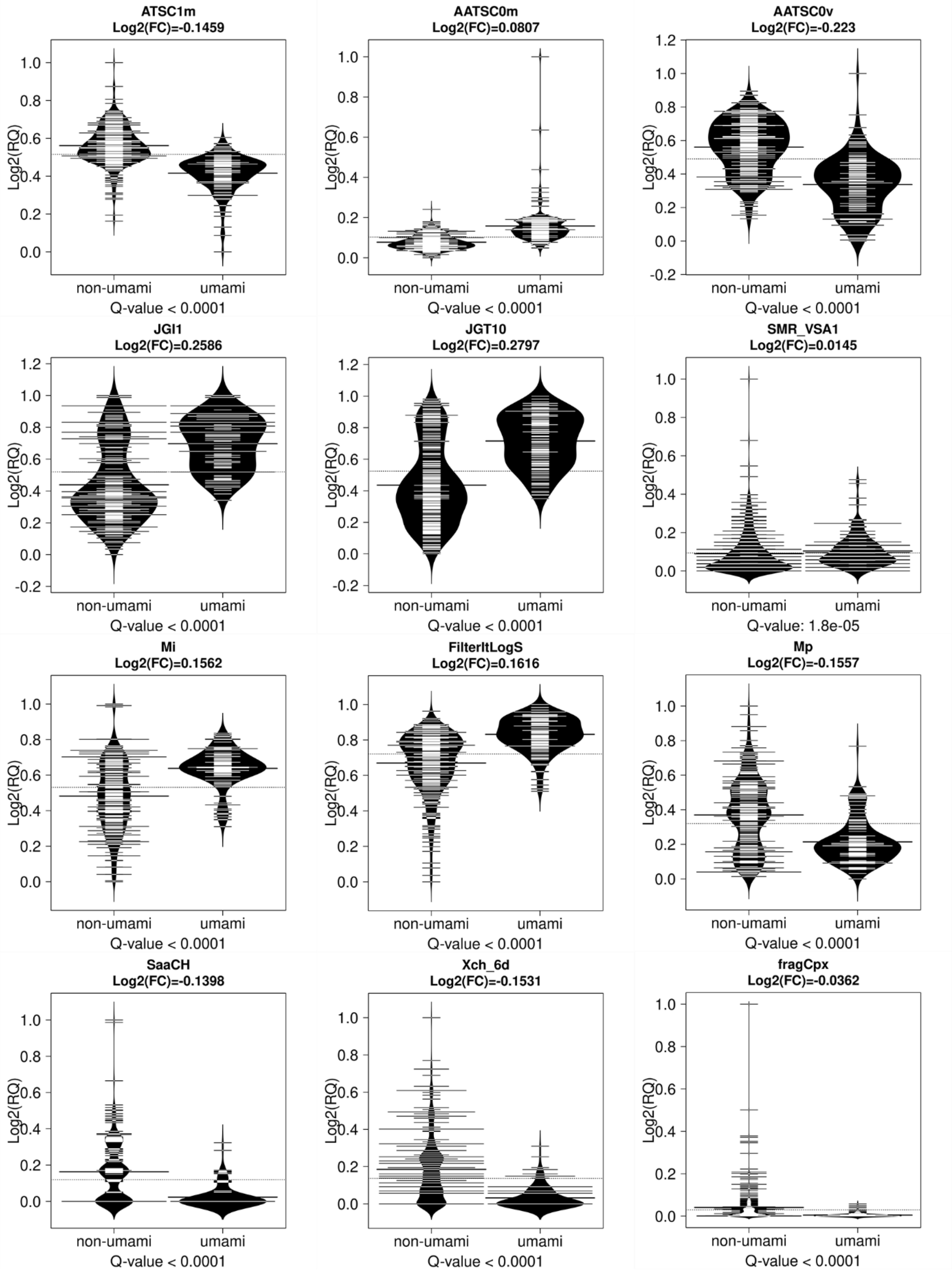

Figure S2. Violin plots showing the distribution of the 12 features in the umami and the non-umami compounds.

Figure S3 represents the hierarchical clustering of the best 12 features, highlighting three major clusters.

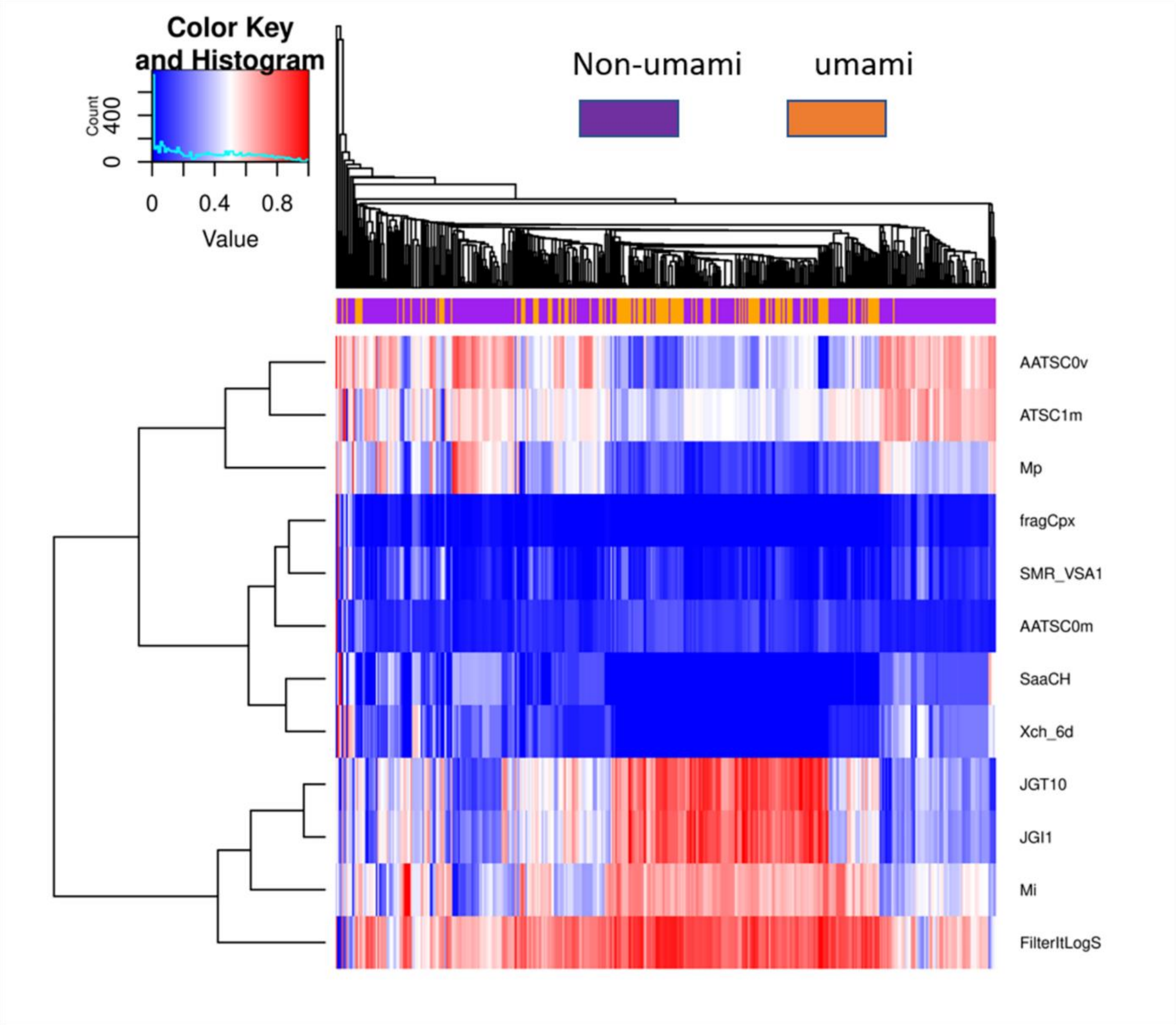

Figure S3. Hierarchical clustering of the selected features reveals 3 groups of features

Discussion

Table S4. Comparison between VirtuousUmami and state-of-the-art umami prediction tools on the VirtuousUmami test set.

	ACC	*Spec*	*Sens*	*F1*	*F2*
**iUmami-SCM**	86.7	93.5	71.4	76.9	73.5
**UMPred-FRL**	88.9	93.5	78.6	81.5	79.7
VirtuousUmami	**87.6**	**91.8**	**78.6**	**79.3**	**81.0**

Results indicate that considered predictors have comparable performance when tested on the VirtuousUmami independent test set. In this context, it is worth mentioning that some of the compounds present in the VirtuousUmami independent test set may come from training sets used to develop iUmami-SCM and UMPred-FRL. This could result in an unfavourable condition for the VirtuousUmami algorithm for which the test set is completely unknown. Despite this potential adverse condition, our algorithm demonstrates a predictive power at least comparable with its predecessors.

Materials and Methods – Data curation

Table S5. Summary of the starting dataset, i.e. the UMP442 database

Class	Number	References
umami	140	Previous literature[3–8] and the BIOPEP-UWM database[9]
non-umami	302	Bitter peptides from BTP640 database[10]

Table S6. Summary of the final dataset used in the present work.

Set	Class	Number
**Training**	umami	240*
	non-umami	240
**Test**	umami	28
	non-umami	62

**Since the non-umami class is oversampled in the training set, we created synthetic data by randomly duplicating some umami compounds to balance the training dataset*
